# Association of smoking and ALK tyrosine-kinase inhibitors on overall survival in treatment-naïve ALK-positive advanced lung adenocarcinoma

**DOI:** 10.3389/fonc.2023.1063695

**Published:** 2023-03-17

**Authors:** Zhe-Rong Zheng, Hsiu-Ying Ku, Kun-Chieh Chen, Chun-Ju Chiang, Chih-Liang Wang, Chih-Yi Chen, Chun-Ming Tsai, Ming-Shyan Huang, Chong-Jen Yu, Jin-Shing Chen, Teh-Ying Chou, Wen-Chung Lee, Chun-Chieh Wang, Tsang-Wu Liu, Jiun-Yi Hsia, Gee-Chen Chang

**Affiliations:** ^1^ Institute of Medicine, Chung Shan Medical University, Taichung, Taiwan; ^2^ Division of Pulmonary Medicine, Department of Internal Medicine, Chung Shan Medical University Hospital, Taichung, Taiwan; ^3^ School of Medicine, Chung Shan Medical University, Taichung, Taiwan; ^4^ National Institute of Cancer Research, National Health Research Institutes, Tainan, Miaoli, Taiwan; ^5^ Institute of Epidemiology and Preventive Medicine, College of Public Health, National Taiwan University, Taipei, Taiwan; ^6^ Taiwan Cancer Registry, Taipei, Taiwan; ^7^ Department of Thoracic Medicine, Chang Gung Memorial Hospital, Taoyuan, Taiwan; ^8^ College of Medicine, Chang Gung University, Taoyuan, Taiwan; ^9^ Division of Thoracic Surgery, Department of Surgery, Chung Shan Medical University Hospital, Taichung, Taiwan; ^10^ Department of Oncology, Taipei Veterans General Hospital, Taipei, Taiwan; ^11^ Division of Pulmonary Medicine, Department of Internal Medicine, E-Da Cancer Hospital, Kaohsiung, Taiwan; ^12^ Department of Internal Medicine, Kaohsiung Medical University Hospital, Kaohsiung, Taiwan; ^13^ School of Medicine, I-Shou University and Kaohsiung Medical University, Kaohsiung, Taiwan; ^14^ Department of Internal Medicine, National Taiwan University Hospital, Taipei, Taiwan; ^15^ College of Medicine, National Taiwan University, Taipei, Taiwan; ^16^ National Taiwan University Hospital Hsinchu Branch, Hsinchu, Taiwan; ^17^ Department of Surgical Oncology, National Taiwan University Cancer Center, Taipei, Taiwan; ^18^ Division of Thoracic Surgery, Department of Surgery, National Taiwan University Hospital, Taipei, Taiwan; ^19^ Graduate Institute of Clinical Medicine, School of Medicine, Taipei Medical University, Taipei, Taiwan; ^20^ Department of Pathology, Taipei Medical University Hospital, Taipei Medical University, Taipei, Taiwan; ^21^ Department of Radiation Oncology, Chang Gung Memorial Hospital-LinKou, Taoyuan, Taiwan; ^22^ Institute of Biomedical Sciences, National Chung Hsing University, Taichung, Taiwan

**Keywords:** lung cancer, TKI - tyrosine kinase inhibitor, smoking, ALK (anaplastic lymphoma kinase), non-small cell lung cancer, ALK non-small cell lung cancer

## Abstract

**Introduction:**

Anaplastic lymphoma kinase (ALK) fusion mutation is more common in younger and never-smoking lung cancer patients. The association of smoking and ALK-tyrosine kinase inhibitors (TKIs) on overall survival (OS) of treatment-naïve ALK-positive advanced lung adenocarcinoma remains unclear in real-world.

**Methods:**

This retrospective study evaluated all 33170 lung adenocarcinoma patients registered in the National Taiwan Cancer Registry from 2017 to 2019, of whom 9575 advanced stage patients had ALK mutation data.

**Results:**

Among the 9575 patients, 650 (6.8%) patients had ALK mutation with the median follow-up survival time 30.97 months (median age, 62 years; 125 [19.2%] were aged ≥75 years; 357 (54.9%) females; 179 (27.5) smokers, 461 (70.9%) never-smokers, 10 (1.5%) with unknown smoking status; and 544 (83.7%) with first-line ALK-TKI treatment). Overall, of 535 patients with known smoking status who received first-line ALK-TKI treatment, never-smokers and smokers had a median OS of 40.7 months (95% confidence interval (CI), 33.1-47.2 months) and 23.5 months (95% CI, 11.5-35.5 months) (P=0.015), respectively. Among never-smokers, those who received first-line ALK-TKI treatment had a median OS of 40.7 months (95% CI, 22.7-57.8 months), while those ALK-TKI not as first-line treatment had a median OS of 31.7 months (95% CI, 15.2-42.8 months) (P=0.23). In smokers, the median OS for these patients was 23.5 months (95% CI, 11.5-35.5 months) and 15.6 months (95% CI, 10.2-21.1 months) (P=0.026), respectively.

**Conclusions and relevance:**

For patients with treatment-naïve advanced lung adenocarcinoma, the ALK test should be performed irrespective of smoking status and age. Smokers had shorter median OS than never-smokers among treatment-naïve-ALK-positive patients with first-line ALK-TKI treatment. Furthermore, smokers not receiving first-line ALK-TKI treatment had inferior OS. Further investigations for the first-line treatment of ALK-positive smoking advanced lung adenocarcinoma patients are needed.

## 1 Introduction

Lung cancer is the leading cause of cancer death worldwide (1, 2). Mutations over several driver genes, such as epidermal growth factor receptor (*EGFR*), echinoderm microtubule-associated protein-like 4-anaplastic lymphoma kinase (*EML4-ALK*) fusion mutations, Kirsten rat sarcoma viral oncogene homolog, and human *EGFR* 2, are known to be involved in the initiation and maintenance of lung adenocarcinoma (3). Different kinds of driver gene mutations result in different clinical characteristics. *EML4-ALK* translocation is more common in younger patients and never-smokers (4, 5). Further, the mutation can be detected in approximately 3-5% of non-small cell lung cancer (NSCLC) patients (6). Several ALK tyrosine kinase inhibitors (TKIs), if administered as first-line treatment, can effectively suppress the oncogenic activity of ALK rearrangement and improve the outcomes of advanced ALK-positive lung cancer patients (7–11). Crizotinib was the first agent approved as it improved the progression-free survival (PFS) compared with platinum-based chemotherapy (10). Subsequently, several next-generation ALK-TKIs including alectinib, brigatinib and lorlatinib showed better PFS and intracranial efficacy compared with crizotinib in treatment-naïve setting (7, 8, 12). Therefore, these were preferred first line therapy in treatment-naïve patients. However, no clinical trials direct compare second- and third- generation ALK-TKIs and no treatment sequence after first-line therapy was suggested. How to make the right choice is based on factors including systemic and intracranial efficacy of the ALK-TKIs, various EML4-ALK variants, mechanisms of resistance as well as the toxicity profile.

Smoking is not only associated with lung cancer incidence, but also influences the efficacy of lung cancer treatment (13). ALK-TKIs as first-line (10, 11, 14) or second-line (15) treatments show similar benefits of PFS in never-smokers and smokers, but overall survival (OS) is immature for most trials (7–11). Meanwhile, the association of smoking and ALK-TKIs on OS of treatment-naïve ALK-positive advanced lung adenocarcinoma patients in the real world remains unclear. Thus, this study aimed to explore the epidemiology, clinical characteristics, and OS of treatment-naïve ALK-positive advanced lung adenocarcinoma patients, focusing on smoking status and ALK-TKIs treatment, using a nationwide cancer registry database in Taiwan.

## 2 Methods

### 2.1 Study design and patients

This retrospective cohort study used data from the National Taiwan Cancer Registry. The database stores standardised records of characteristics and clinical information of all cancer patients in Taiwan since 1979 (16–19). Detailed information on the smoking status and first-line treatment modalities and regimens for lung cancer patients has been recorded in the database starting since 2011, and ALK mutation data were added since 1 January 2017.

The current study analysed the data of treatment-naïve ALK-positive advanced lung adenocarcinoma patients recorded in the database between 01 January 2017 and 31 December 2019. In Taiwan, as Ventana immunohistochemistry (IHC) ALK (D5F3) detection test in lung adenocarcinoma was reimbursed by National Health Insurance Administration (NHIA), so most of the ALK gene fusion was detected by this method. The inclusion criterion was cytological or pathological evidence of lung cancer and a clear classification of adenocarcinoma subtype. The National Taiwan Cancer Registry did not have ALK fusion data in squamous cell carcinoma of lung. The data were not available in this study. The association of smoking status, ALK-TKIs treatment, and clinical characteristics with OS was evaluated in ALK-positive advanced lung adenocarcinoma patients. The survival follow-up was until 31 December 2020.

The study protocol was approved by the Institutional Review Board of the National Health Research Institutes in Taiwan (approval number: EC1080506-E). The Strengthening the Reporting of Observational Studies in Epidemiology (STROBE) reporting guideline for observational studies was used to report the findings of this article (20).

### 2.2 Data collection

Data included age at diagnosis, sex, histological types, tumour stage, smoking status, Eastern Cooperative Oncology Group performance status (ECOG PS), ALK mutation status, and survival status. Patients were classified as never-smokers if they had never smoked in their lifetime; otherwise, they were classified as smokers. Before 2018, lung cancer staging in the registry was according to the 7^th^ edition of the American Joint Committee on Cancer (AJCC) staging system; and thereafter, according to the AJCC 8^th^ edition (21, 22).

### 2.3 Statistical analysis

OS was calculated from the date of diagnosis to the date of death or the last follow-up. Survival status, which was evaluated using the National Death Certificates Database from the Department of Statistics, Ministry of Health and Welfare, Taiwan, was updated until 31 December 2020. Records were excluded if the date of death was unknown. The chi-square test was used to evaluate the association between categorical variables. OS curves were generated using the Kaplan–Meier method and compared using the log-rank test. The association between clinicopathological variables and outcomes was assessed using Cox proportional hazard regression models. Hazard ratios (HR) and 95% confidence intervals (CIs) were calculated using univariate and multivariable models. A two-sided P < 0.05 was considered statistically significant. All statistical analyses were performed using SPSS version 22.0 (SPSS Inc., Chicago, IL, USA) software.

## 3 Results

A total of 46,897 patients were diagnosed with lung cancer during the study period, and 33170 (70.7%) had lung adenocarcinoma (**Supplement Table 1A**). Among patients with advanced stage (stages IIIB to IV) lung adenocarcinoma, 9575 had ALK mutation data; of these, 650 were ALK mutation positive with the median follow-up survival time 30.97 months. The 650 patients median age was 62 years, 125 (19.2%) patients were aged ≥75 years, 357 (54.9%) were female, and 640 and 10 (1.5%) had known and unknown smoking status, respectively. Overall, 179 (27.5) and 461 (70.9%) patients were smokers and never-smokers; 554 (85.2%) had ECOG PS 0-2. There were 592 patients (91.1%) with stage IV disease, and 313 (48.2%) had primary tumours in the upper lobes. Five hundred forty-four patients (83.7%) received ALK-TKI as first-line treatment (**Supplement Table 1B**).

Among the 9575, 5742, 3725, and 108 patients with known mutation data, never-smokers, smokers, and unknown smoking status, the ALK mutation rates were 6.8% (n=650), 8.0% (n=461), 4.8% (n=179), and 9.3% (n=10), respectively (**Table 1**). There were significantly more females among never-smokers (72.0%), whereas there were more males (90.5%) among smokers (P<0.001). Meanwhile, there were no differences in age distribution, ECOG PS, stage, primary tumour location, and use of first-line ALK-TKI treatment between never-smokers and smokers (**Table 2**). Concerning OS among ALK mutation-positive patients (n=640), univariate and multivariable analyses showed that those aged <65 years, never-smokers, those with better ECOG PS, and with stage IIIB or IIIC disease had significantly better OS outcomes. OS did not differ according to sex and use of first-line ALK-TKI treatment. Primary tumour location over the lower lobe had better OS in univariate, but no difference in multivariable analyses (**Supplement Table 2**).

**Table 1:** ALK-positive rates.

	Stage IIIB/IIIC		Stage IV		Total (%)	
	n	%	n	%	n	%
Never-smokers (n=5742)						
ALK mutation (+)	39	(12.0%)	422	(7.8%)	461	(8.0%)
ALK mutation (-)	285	(88.0%)	4996	(92.2%)	5281	(92.0%)
Smokers (n=3725)						
ALK mutation (+)	17	(4.7%)	162	(4.8%)	179	(4.8%)
ALK mutation (-)	341	(95.3%)	3205	(95.2%)	3546	(95.2%)
Unknown smoking status (n=108)						
ALK mutation (+)	2	(20.0%)	8	(8.2%)	10	(9.3%)
ALK mutation (-)	8	(80.0%)	90	(91.8%)	98	(90.7%)
Total (n=9575)						
ALK mutation (+)	58	(8.4%)	592	(6.7%)	650	(6.8%)
ALK mutation (-)	634	(91.6%)	8291	(93.3%)	8925	(93.2%)

AJCC 7^TH^ edition before 2018.

AJCC 8^th^ edition since 2018.

ALK, anaplastic lymphoma kinase.

**Table 2:** Characteristics of the patients with known smoking status.

Patient characteristics	Total (n=640, 100%)		Never-smokers (n=461, 72.0%)		Smokers (n=179, 28.0%)		P value
	n	%	n	%	n	%	
Age, years							
<65	358	55.9%	250	54.2%	108	60.3%	0.36
65-74	158	24.7%	117	25.4%	41	22.9%	
≥75	124	19.4%	94	20.4%	30	16.8%	
Sex							
Male	291	45.5%	129	28.0%	162	90.5%	<0.001
Female	349	54.5%	332	72.0%	17	9.5%	
ECOG performance status							
0-2	547	85.5%	393	85.2%	154	86.0%	0.75
3-4	43	6.7%	33	7.2%	10	5.6%	
Unknown	50	7.8%	35	7.6%	15	8.4%	
Stage							
IIIB or IIIC	56	8.8%	39	8.5%	17	9.5%	0.68
IV	584	91.2%	422	91.5%	162	90.5%	
Tumour location							
Upper lobes	311	48.6%	215	46.6%	96	53.6%	0.26
Lower lobes	290	45.3%	218	47.3%	72	40.2%	
Others	39	6.1%	28	6.1%	11	6.1%	
First-line ALK TKI							
Yes	535	83.6%	388	84.2%	147	82.1%	0.80
No	105	16.4%	73	15.8%	32	17.9%	

AJCC 7^TH^ edition before 2018.

AJCC 8^th^ edition since 2018.

Upper lobe includes the right middle lobe; others include bilateral lung lesions, trachea lesions, and main bronchus lesions.

With respect to OS according to smoking status, univariate analysis showed that in never-smokers with aged <75 years, male sex, ECOG PS 0-2, stage IV disease, primary tumour location over the upper lobe, and ALK-TKI treatment had better OS than did their smoker counterparts (**Table 3A**). Meanwhile, OS did not differ between smokers and never-smokers among those aged ≥75 years, female sex, ECOG PS 3-4, stage IIIB or IIIC disease, and primary tumours over lower lobes. **Table 3B** shows the multivariable analysis of influencing factors of OS in smokers and never-smokers were performed separately. In never-smokers with ALK mutation, age <65 years, and ECOG PS 0-2 had better OS, while there were no differences in OS according to sex, disease stage, primary tumour location, and first-line ALK-TKI treatment. In smokers, ALK-positive patients aged <65 years and with first-line ALK-TKI treatment had better OS, while there were no differences in OS according to sex, ECOG PS status, disease stage, and primary tumour location.

**Table 3A:** Univariate analysis of overall survival in different subgroups between smokers and never-smokers.

	Smokers vs. Never-smokers			P-value
	HR	(95% CI)		
Age, years				
<65	1.531	1.066	2.200	0.021
65-74	2.121	1.307	3.440	0.002
≥75	1.538	0.967	2.446	0.069
Sex				
Male	1.805	1.259	2.586	0.001
Female	1.584	0.833	3.013	0.161
ECOG performance status				
0-2	1.524	1.157	2.007	0.003
3-4	1.175	0.557	2.477	0.672
Unknown	1.849	0.843	4.054	0.125
Stage				
IIIB or IIIC	1.709	0.687	4.255	0.249
IV	1.501	1.165	1.935	0.002
Tumour location				
Upper lobes	1.851	1.335	2.567	<0.001
Lower lobes	1.226	0.820	1.833	0.321
Others	0.684	0.222	2.105	0.510
First-line ALK TKI				
Yes	1.409	1.068	1.860	0.015
No	1.501	1.165	1.935	0.023

**Table 3B:** Multivariable analysis of influencing factors of overall survival in smokers and never-smokers.

Patient characteristics	Never-smokers				Smokers			
	HR	(95%CI)		P value	HR	(95%CI)		P value
Age, years								
<65	1.0 (reference)				1.0 (reference)			
65-74	1.464	1.016	2.109	0.041	2.256	1.322	3.849	0.003
≥75	2.916	2.043	4.162	<0.001	4.338	2.512	7.491	0.000
Sex								
Male	1.0 (reference)				1.0 (reference)			
Female	1.159	0.822	1.634	0.401	0.843	0.412	1.723	0.639
ECOG performance status								
0-2	1.0 (reference)				1.0 (reference)			
3-4	3.019	1.890	4.825	<0.001	2.017	0.980	4.153	0.057
Unknown	1.533	0.888	2.644	0.125	2.039	0.979	4.249	0.057
Stage								
IIIB or IIIC	1.0 (reference)				1.0 (reference)			
IV	1.496	0.806	2.779	0.202	1.962	0.925	4.160	0.079
Tumour location								
Upper lobe	1.0 (reference)				1.0 (reference)			
Lower lobe	1.000	0.743	1.347	1.000	0.708	0.442	1.136	0.153
Others	0.857	0.461	1.590	0.624	0.658	0.229	1.892	0.438
First-line ALK TKI								
No	1.0 (reference)				1.0 (reference)			
Yes	1.014	0.627	1.638	0.956	0.392	0.204	0.752	0.005

HR, hazard ratio; CI, confidence interval.

AJCC 7^TH^ edition before 2018.

AJCC 8^th^ edition since 2018.

Upper lobe includes the right middle lobe; others include bilateral lung lesions, trachea lesions, and main bronchus lesions.

**Figure 1:**
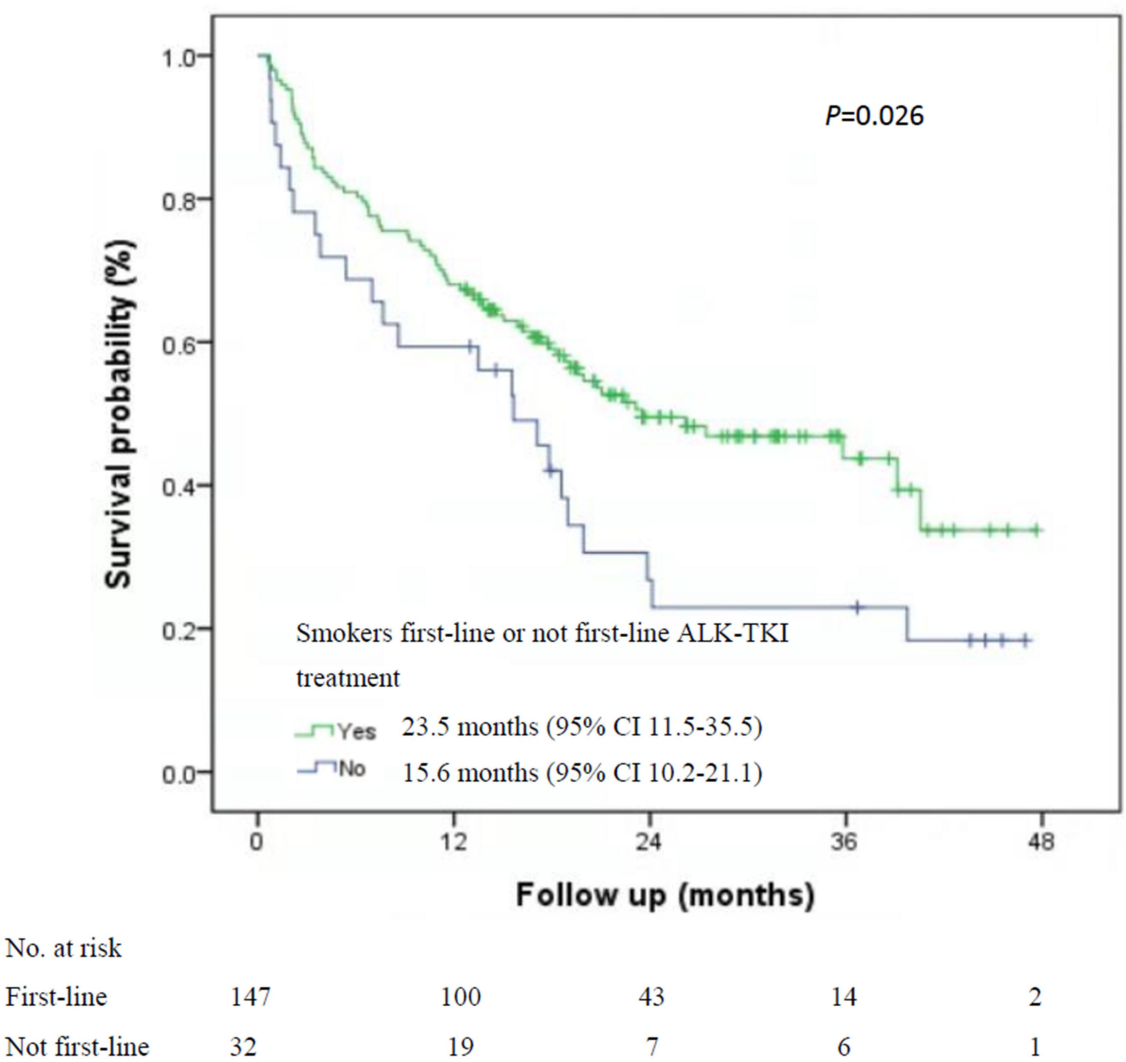
Kaplan-Meier curves of overall survival by smoking status among the 535 patients with first-line ALK-TKI treatment. ALK-TKI, anaplastic lymphoma kinase tyrosine kinase inhibitors.

**Figure 2:**
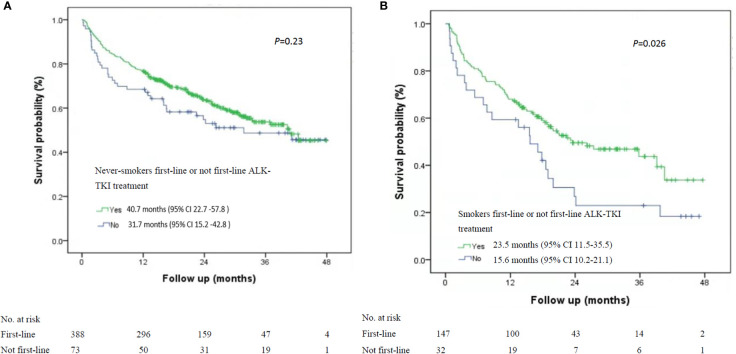
Kaplan-Meier curves of overall survival stratified by smoking status and ALK-TKI treatment line in ALK-positive patients. **(A)** Never-smokers and first-line or not first-line ALK-TKI treatment (n=461). **(B)** Smokers and first-line or not first-line ALK TKI treatment (n=179). ALK-TKI, anaplastic lymphoma kinase tyrosine kinase inhibitors.

Among the 535 patients with known smoking status who received first-line ALK-TKI treatment, never-smokers had higher median OS than did smokers (40.7 months; 95% CI, 33.1-47.2 months vs. 23.5 months, 95% CI, 11.5-35.5 months; P=0.015; **Figure 1**). For OS according to the treatment of ALT-TKI among never-smokers, those who received first-line ALK-TKI treatment had longer median OS than those who did not receive ALK-TKI in the first-line setting, but the difference was not significant (40.7 months; 95% CI, 22.7-57.8 months vs. 31.7 months; 95% CI, 15.2-42.8 months; P=0.23; **Figure 2A**). Meanwhile, for smokers, those who received first-line ALK-TKI treatment had significantly higher median OS than those who did not receive ALK-TKI in the first-line setting (23.5 months; 95% CI, 11.5-35.5 months vs. 15.6 months; 95% CI, 10.2-21.1 months; P=0.026; **Figure 2B**).

## 4 Discussion

The association of smoking and ALK-TKIs on OS in treatment-naïve ALK-positive advanced lung adenocarcinoma patients is yet to be elucidated. This study using 2017-2019 data from the National Taiwan Cancer Registry database found that among patients who received first-line ALK-TKI treatment, the median OS was shorter among smokers than among never-smokers. Furthermore, in smokers, patients with ALK mutation who did not receive ALK-TKI as the first-line treatment had inferior median OS. These findings support the urgent need to consider new first-line treatment modalities for ALK-positive advanced lung adenocarcinoma patients who are smokers. The Taiwan nationwide database allows for the evaluation of OS according to smoking status and ALK-TKIs treatment. Also, it provides detailed information about clinical features that can influence OS.

*EML4-ALK* translocation is more common in never-smokers and younger patients. However, among the 650 patients in this study, approximately 30% of patients were current or former smokers, and 20% were aged >75 years. As such, it is necessary that all patients are evaluated for ALK gene rearrangements, irrespective of their smoking status and age. In Taiwan, three evaluation methods were allowed for ALK-TKIs reimbursement. The most commonly used is a fully automated immunohistochemistry assay (Ventana IHC, Ventana, Tucson, AZ) with the prediluted Ventana anti-ALK (D5F3) Rabbit monoclonal primary antibody as previously described (4). The other two methods, namely, next-generation sequencing and fluorescence *in situ* hybridization, are used less frequently.

Randomised clinical trials (23–25) of lung cancer patients with EGFR-sensitive mutation showed that first-line treatment with EGFR-TKIs achieved similar PFS benefits to chemotherapy in smokers and never-smokers. Meanwhile, a meta-analysis showed that never-smokers had better PFS benefits than did smokers (26). Several trials showed similar PFS benefits over chemotherapy using ALK-TKIs between smokers and never-smokers for patients with treatment-naïve advanced stage ALK mutation-positive lung cancer (10, 11, 14). However, in ALK mutation-positive patients, there are no clinical trial data on OS differences between smokers and never-smokers.

A real-world study evaluated 121 stage IV ALK mutation-positive NSCLC patients diagnosed between 2011 and 2016 and showed that never-smoking was the only independent prognostic factor associated with better OS (HR: 0.499, 95% CI: 0.265-0.941, P=0.032). The use of alectinib or lorlatinib in any treatment line improved OS (P=0.022) (27). Similar findings were observed in the current study, wherein crizotinib was the most common first-line treatment for ALK mutation-positive lung cancer patients, which may be explained by the reimbursement guidelines on ALK-TKIs by the Taiwan National Health Insurance Administration (**Supplementary Table 3**). The reimbursement for crizotinib use as a first-line treatment began in November 2017, but it has been available as a second-line treatment since 2015. A proportion of the patients had used crizotinib as the first-line treatment before 2017. This explains the high percentage of first-line ALK-TKI use (83.6%) in this study. The same observations were noted for ceritinib and alectinib use.

The current study found that among patients with first-line ALK-TKI treatment, never-smokers had better median OS than smokers. In smokers, OS was significantly better in ALK-positive patients who received first-line ALK-TKI treatment than those who did not receive. This could be because first-line ALK-TKI treatment was less optimal in smokers than in never-smokers; however, clinical trials showed that it is still better than chemotherapy in smokers (10, 11, 14).

The possible mechanisms about the inferior OS in smoking ALK-positive patients remain unclear. In our previous study, the presence of ALK V3a/b subtype independently predicted a worse overall survival in patients receiving ALK inhibitors, however, the incidences of V3 subtype were similar between smokers and never-smokers (5). Smokers suffered more mutations than never-smokers and this could complicate the drug efficacy. Among smokers, TP53 mutation is the most common (28). There is a high rate of TP53 comutation in ALK-positive lung cancer patients, which has shown a significantly worse prognosis (29, 30).Additional studies are required to clarify the potential mechanisms.

There are limitations to this study. OS data from the PROFILE-1014 trial differ based on the access to subsequent ALK-TKIs after crizotinib failure, with a 5-year survival rate of 75% vs. 28% in patients who did and did not receive subsequent ALK-TKI, respectively (31, 32). First, we did not have detailed data on treatment after failure of first-line treatment. However, as ALK-TKIs were subsequently reimbursed in Taiwan (first-line crizotinib began in November 2017 and second-generation ALK-TKIs as the second line since September 2017), most patients who failed first-line ALK-TKIs treatment in the study would have had the chance to receive ceritinib or alectinib. Some even received brigatinib or lorlatinib in subsequent treatments. Second, our ALK mutation-positive patients had shorter OS than those reported in clinical trials. This could be because not all patients received alectinib or lorlatinib as subsequent treatment. Lorlatinib was not reimbursed as the second line until June 2020 and was limited to disease progression to the brain after ceritinib or alectinib failure. The use of more ALK-TKI lines and the use of alectinib or lorlatinib in any treatment line are positively correlated with OS (27). Other limitations include a high proportion of patients older than 75 years and with poor PS.

## 5 Conclusion

For patients with treatment-naïve advanced lung adenocarcinoma, the ALK test should be performed irrespective of smoking status and age. For ALK mutation-positive patients with first-line ALK-TKI treatment, smokers have shorter median OS than never-smokers. In smokers, the survival would be inferior if they did not receive first-line ALK-TKIs treatment. Further investigations for the first-line treatment of ALK-positive smoking advanced lung adenocarcinoma patients are needed.

## Data availability statement

The original contributions presented in the study are included in the article/**Supplementary Material**. Further inquiries can be directed to the corresponding authors.

## Ethics statement

The study protocol was approved by the Institutional Review Board of the National Health Research Institutes in Taiwan (approval number: EC1080506-E). Written informed consent to participate in this study was provided by the participants’ legal guardian/next of kin.

## Author contributions 

G-CC and J-YH had full access to all of the data in the study and take responsibility for the integrity of the data and the accuracy of the data analysis. Z-RZ, H-YK, and K-CC contributed equally to this work as the co-first authors. G-CC and J-YH contributed equally to this work. Concept and design: Z-RZ, H-YK, K-CC, J-YH, and G-CC. Acquisition, analysis, or interpretation of data: All authors. Drafting of the manuscript: Z-RZ, H-YK, K-CC, J-YH, and G-CC. Critical revision of the manuscript for important intellectual content: C-JC, Chen, W-CL, T-WL, J-YH, and G-CC. Statistical analysis: Z-RZ, H-YK, C-JC, and G-CC. Obtained funding: T-WL and G-CC. Administrative, technical, or material support: Z-RZ, H-YK, Chen, C-JC, W-CL, T-WL, J-YH, and G-CC. Supervision: RZ, H-YK, Chen, J-YH, and G-CC. All authors contributed to the article and approved the submitted version.
