# Supplementary material for: Association of smoking and ALK tyrosine-kinase inhibitors on overall survival in treatment-naïve ALK-positive advanced lung adenocarcinoma
Source: Front Oncol. 2023 Mar 17;13:1063695. doi: 10.3389/fonc.2023.1063695 (PMC10064125; doi:10.3389/fonc.2023.1063695)
Supplement: Supplementary file 1 [file Table_1.docx]

**Supplement Content**

Supplement Table 1A. Total number of lung cancer cases and subtypes

| Year | Total | Adenocarcinoma | SCC | SCLC | Others |
| --- | --- | --- | --- | --- | --- |
| 2017 | 14846 | 10273 (69.2%) | 1920 (12.9%) | 1010 (6.8%) | 1643 (11.1%) |
| 2018 | 15818 | 11303 (71.5%) | 1909 (12.1%) | 997 (6.3%) | 1609 (10.2%) |
| 2019 | 16233 | 11594 (71.4%) | 1933 (11.9%) | 1084 (6.7%) | 1622 (10.0%) |
| Total | 46897 | 33170 (70.7%) | 5762 (12.3%) | 3091 (6.6%) | 4874 (10.4%) |

SCC, squamous cell carcinoma; SCLC, small-cell lung cancer

Supplement Table 1B. Patient characteristics (N=650)

| Characteristic | Value |
| --- | --- |
| Age (yrs) |  |
| Mean±SD | 61.2±13.8 |
| Median (IQR, 25%-75%) | 62 (51-71) |
| Range | 25-92 |
| Age (years), n (%) |  |
| <65 | 364 (56.0) |
| 65-74 | 161 (24.8) |
| ≥75 | 125 (19.2) |
| Sex, n (%) |  |
| Male | 293 (45.1) |
| Female | 357 (54.9) |
| Smoking status, n (%) |  |
| Never-smokers | 461 (70.9) |
| Smokers | 179 (27.5) |
| Unknown | 10 (1.5) |
| ECOG PS, n (%) |  |
| 0-2 | 554 (85.2) |
| 3-4 | 43 (6.6) |
| Unknown | 53 (8.2) |
| Stage, n (%) |  |
| IIIB/ IIIC | 58 (8.9) |
| IV | 592 (91.1) |
| Tumour location, n (%) |  |
| Upper lobe | 313 (48.2) |
| Lower lobe | 296 (45.5) |
| Others | 41 (6.3) |
| First-line ALK TKI, n (%) | |
| Yes | 544 (83.7) |
| No | 106 (16.3) |

AJCC 7^TH^ edition before 2018

AJCC 8^th^ edition since 2018

Upper lobe includes the right middle lobe; Others include bilateral lung lesions, trachea lesions, and main bronchus lesions

Supplement Table 2. Univariate and multivariable analysis of influencing factors of overall survival

| Patient characteristics | **Univariable** | | | | **Multivariable** | | | |
| --- | --- | --- | --- | --- | --- | --- | --- | --- |
|  | HR | Lower | Upper | P value | HR | Lower | Upper | P value |
| Age, years |  |  |  |  |  |  |  |  |
| <65 | 1 |  |  |  | 1 |  |  |  |
| 65-74 | 1.52 | 1.14 | 2.03 | 0.01 | 1.68 | 1.25 | 2.25 | <0.001 |
| ≥75 | 3.50 | 2.66 | 4.59 | <0.001 | 3.12 | 2.34 | 4.17 | <0.001 |
| Sex |  |  |  |  |  |  |  |  |
| Male | 1 |  |  |  | 1 |  |  |  |
| Female | 0.95 | 0.75 | 1.20 | 0.67 | 1.11 | 0.83 | 1.50 | 0.48 |
| Smoking status |  |  |  |  |  |  |  |  |
| Never-smokers | 1 |  |  |  | 1 |  |  |  |
| Smokers | 1.50 | 1.17 | 1.91 | <0.001 | 1.81 | 1.32 | 2.48 | <0.001 |
| ECOG PS |  |  |  |  |  |  |  |  |
| 0-2 | 1 |  |  |  | 1 |  |  |  |
| 3-4 | 4.27 | 2.99 | 6.09 | <0.001 | 2.71 | 1.87 | 3.94 | <0.001 |
| Unknown | 1.35 | 0.90 | 2.03 | 0.15 | 1.53 | 1.01 | 2.32 | 0.04 |
| Stage |  |  |  |  |  |  |  |  |
| IIIB or IIIC | 1 |  |  |  | 1 |  |  |  |
| IV | 1.64 | 1.03 | 2.62 | 0.04 | 1.71 | 1.07 | 2.74 | 0.03 |
| Tumour location |  |  |  |  |  |  |  |  |
| Upper lobe | 1 |  |  |  | 1 |  |  |  |
| Lower lobe | 0.77 | 0.60 | 0.98 | 0.03 | 0.88 | 0.69 | 1.12 | 0.30 |
| Others | 0.76 | 0.46 | 1.25 | 0.27 | 0.72 | 0.43 | 1.22 | 0.22 |
| First-line ALK-TKI |  |  |  |  |  |  |  |  |
| Yes | 1 |  |  |  | 1 |  |  |  |
| No | 0.71 | 0.53 | 0.94 | 0.02 | 0.77 | 0.57 | 1.04 | 0.09 |

AJCC 7^TH^ edition before 2018

AJCC 8^th^ edition since 2018

Upper lobe includes the right middle lobe; Others include bilateral lung lesions, trachea lesions, and main bronchus lesions

Supplement Table 3. Timeline of ALK-TKI reimbursement by the Taiwan National Health Insurance Administration

| Drugs | Second line | First line |
| --- | --- | --- |
| Crizotinib | September 2015 | November 2017 |
| Ceritinib | September 2017 | July 2019 |
| Alectinib | November 2017 | December 2019 |
| Brigatinib | August 2020 |  |
| Lorlatinib | June 2020 only in brain progression with metastasis after ceritinib or alectinib |  |
